# How Do Soccer Players Adjust Their Activity in Team Coordination? An Enactive Phenomenological Analysis

**DOI:** 10.3389/fpsyg.2017.00854

**Published:** 2017-05-26

**Authors:** Vincent Gesbert, Annick Durny, Denis Hauw

**Affiliations:** ^1^Institute of Sport Sciences, Faculty of Social and Political Sciences, University of LausanneLausanne, Switzerland; ^2^“Movement Sport Health” Laboratory (EA1234), University of Rennes 2Rennes, France

**Keywords:** enactive approach, phenomenological data, elicitation interviews, interpersonal coordination, indirect interpersonal coordination, soccer, collective body memory

## Abstract

This study examined how individual team members adjust their activity to the needs for collective behavior. To do so, we used an enactive phenomenological approach and explored how soccer players' lived experiences were linked to the active regulation of team coordination during eight offensive transition situations. These situations were defined by the shift from defensive to offensive play following a change in ball possession. We collected phenomenological data, which were processed in four steps. First, we reconstructed the diachronic and synchronic dynamics of the players' lived experiences across these situations in order to identify the units of their activity. Second, we connected each player's units of activity side-by-side in chronological order in order to identify the collective units. Each connection was viewed as a collective regulation mode corresponding to which and how individual units were linked at a given moment. Third, we clustered each collective unit using the related objectives within three modes of regulation—local (L), global (G), and mixed (M). Fourth, we compared the occurrences of these modes in relation to the observable key moments in the situations in order to identify typical patterns. The results indicated four patterns of collective regulation modes. Two distinct patterns were identified without ball possession: reorganize the play formation (G and M) and adapt to the actions of putting pressure on the ball carrier (M). Once the ball was recovered, two additional patterns emerged: be available to get the ball out of the recovery zone (L) and shoot for the goal (L and M). These results suggest that team coordination is a fluctuating phenomenon that can be described through the more or less predictable chaining between these patterns. They also highlight that team coordination is supported by several modes of regulation, including our proposal of a new mode of interpersonal regulation. We conclude that future research should investigate the effect of training on the enaction of this mode in competition.

## Introduction

We often take delight in following a fast counterattack in a soccer game, listening to a string quartet, or watching a dance troupe improvising: the wonder is how the multiple social agents manage to coordinate their actions so quickly and suitably. Team performances require the coordinated contributions of two or more members working interdependently to achieve a common objective (e.g., Salas et al., 1992). The key word is coordinated, as this is what determines the result. But for sports psychologists, understanding exactly how team members' actions are successfully coordinated has remained a challenge (e.g., Blickensderfer et al., 2010; Bourbousson et al., 2012; Travassos et al., 2012).

A recent review identified three main theoretical perspectives to explain interpersonal coordination (see Araújo and Bourbousson, 2016). The first is the social-cognitive perspective (e.g., Eccles and Tenenbaum, 2004; Reimer et al., 2006; Blickensderfer et al., 2010), which assumes that through practice and experience team members develop mental representations of the performance environment (also depicted as mental models or knowledge) within which the team members regulate their behaviors to achieve high performances (e.g., Eccles and Tran Turner, 2014). For instance, these mental representations allow team members to predict events or understand the operations being undertaken by the other team members with whom they are interacting (Blickensderfer et al., 2010). According to this approach, to achieve team coordination, a subset of each team member's mental representations must be similar to at least a subset of the mental representations of the other team members, such that each team member can form clear expectations about the others' actions (e.g., Eccles and Tenenbaum, 2004). They then coordinate by adapting to the dynamic changes in the competitive performance environment and by selecting appropriate goal-directed actions to execute at appropriate times (Eccles, 2010).

The second is the ecological dynamics perspective (e.g., Travassos et al., 2012; Silva et al., 2013; Passos et al., 2016), according to which the player's activity is not based on mental representations stored in memory but rather on the perception of surrounding informational constraints. For example, the perception of a basketball defender's most advanced foot might prompt an attacker to drive the attack to that side (Esteves et al., 2011). These surrounding constraints provide players with direct possibilities for acting within the performance environment. The players thus regulate their behaviors through the perception and use of these affordances. At the interpersonal level, through practice, players can become perceptually attuned to the affordances *of* others (i.e., what actions another person affords the perceiver) and the affordances *for* others (i.e., what actions are possible for another person) during competitive performance (Fajen et al., 2008). This allows them to undertake more efficient actions by functionally adjusting their behaviors to those of their teammates and opponents.

The third is the enactive perspective (e.g., Poizat et al., 2009; Bourbousson et al., 2012; Gesbert and Durny, 2017), according to which a team member's activity is based on the process of making meaning. By acting in direction of the other players, he/she feels sensations and makes sense of the players' behaviors that allows him/her to develop a higher order understanding of the situation. Based on what is relevant to the team member in relation to his activity, he/she will be more or less attuned to environmental information. For instance, in a defensive phase, four basketball players may focus on their direct opponent, whereas the fifth thinks that this opponent is not dangerous and chooses instead to observe the game (see Bourbousson et al., 2012). Players actively and asymmetrically regulate the conditions of their exchanges with the environment (e.g., Barandiaran et al., 2009; Froese and Di Paolo, 2011)—they look for and select what is relevant for them to act in the environment. At the interpersonal level, this means that team coordination is dynamically achieved in real time and cannot be prescribed by previous shared knowledge. The study of team coordination phenomena from this perspective refers to the extent to which individual activities contribute to or perturb the activity of others. It notably implies exploring how the meaning that each team member builds in her activity corroborates the meanings simultaneously built by the teammates (i.e., participatory sense making). For instance, in the study of Bourbousson et al. (2012), four players share meanings about the monitoring of their direct opponent but not the fifth player. Despite the recent advances, this conceptualization remains relatively neglected, as noted by Bourbousson and Fortes-Bourbousson (2016). In general, the objective is to determine how individual team members adjust their dynamic involvement in team coordination online and how the other team members simultaneously join in. The involvement means the concerns enacted by each player at a given moment, that is to say what he/she wants to do at a given moment. In the present study, we sought to address these research questions by adopting an enactive approach.

A first study has addressed these questions using an intermediary methodology. For instance, Millar et al. (2013) studied how interpersonal coordination was achieved and maintained in two-person rowing boats by interesting to the experiential knowledge built and used by expert rowers to coordinate during race. They conducted semi-structured interviews (i.e., qualitative methodology) with nine expert rowers and paid close attention to the perceptual information underlying the interpersonal coordination and how the information were used. Their results showed that the expert rowers coordinated their actions without taking each other into account, but rather by being attuned to variations in the boat speed. The authors thus developed the notion of extrapersonal coordination to describe how two rowers manage to achieve tight coordination by articulating their respective activities around an indicator in the situation (i.e., the variation in boat speed).

More recently, other studies have also addressed these questions using an enactive methodology. For instance, R'Kiouak et al. (2016) investigated how two coxless rowers experienced the effectiveness of their joint action during a race. The authors conducted individual self-confrontation interviews with each rower post-race to collect phenomenological data. From these data, the authors reconstructed the dynamics of the lived experience of each rower during the complete race by identifying the chaining of experience units across time. Each of these units is composed of six elements of meaning: current action (i.e., physical action or an interpretative act), involvement (i.e., the individual's concern at a given moment), expectations, prior mobilized knowledge that is relevant to the current situation, perception (i.e., elements of the situation significant to the individual at a given moment) and refashioned knowledge. A detailed examination of these elements of meaning then allowed them to characterize how each rower experienced the joint action effectiveness. Three typical modes of experiencing joint action effectiveness were characterized (i.e., meaningless, effective, and detrimental). The authors then synchronized the rowers' typical experiences in order to examine how the rowers simultaneously and similarly experienced the effectiveness of their joint action during the ongoing performance. Their results indicated that the rowers could experience the joint effectiveness of their joint action as detrimental, effective or divergent. This highlighted the use of an interpersonal regulation mode based on direct co-regulation between the rowers. But their results also indicated that the rowers could not have a meaningful experience of their joint action (i.e., they did not pay attention to the joint action at the level of their activity). This result shed light on the use of an extrapersonal regulation mode based on the adjustment of the rowers' movements in response to information from the boat and the reaction of the water which rowers were attuned. Their results thus indicated that interpersonal coordination was not the constant focus of the rowers' active adaptations. While acting on their oar, the rowers were particularly able to adjust their movements in response to the boat information and the reaction of the water, which allowed them to respond similarly (i.e., extrapersonal regulation mode). The authors then asked several research questions: (a) How might these two modes of regulation co-occur during a given ongoing joint action? (b) What setting characteristics are propitious for one of these regulation modes to emerge? and (c) How actors switch dynamically from one regulation mode to another during an unfolding joint action?

The questions were again raised in the work of Bourbousson and Fortes-Bourbousson (2016), who also highlighted the limited number of studies investigating how team members actively adjust their interpersonal coordination in real time. Although the studies in the sports sciences on the regulation modes enacted by team members have essentially dealt with the rowing dyad, one study investigated how basketball players heed their teammates in the first 10 min of a championship match (Bourbousson et al., 2010). To do so, the researchers filmed a match and then conducted individual self-confrontation interviews with each player. These interviews provided verbalization data on the teammates that each player took into account at a given instant in order to act. Their results showed that, at the level of activity that was meaningful for them, the basketball players most often took a single teammate into account. In cases of one-on-one play, however, sometimes no teammate was taken into consideration. The results also revealed that only 13% of the coordinations were reciprocal and that therefore the network of connections was for the most part built of one-directional cognitive links. These results cast doubt on the long-held assumption of the need for a co-regulation mode among team members in order to coordinate. According to Bourbousson et al. (Bourbousson et al., 2010; Bourbousson and Fortes-Bourbousson, 2016), these co-regulation modes may occur only between certain teammates, with the team then functioning on the basis of these few coordination links. The results also raised questions about the regulation modes enacted by members in the case of bigger social systems (i.e., a team sport).

This study sought to respond to these questions by investigating the regulation modes enacted by soccer team members in order to play with tight coordination during a match. In the research cited above, special attention was given to how the agents experienced their ongoing activity and regulated team coordination. This has been one of the pillars of the enactive approach since Varela's work (e.g., Varela et al., 1991; Di Paolo et al., 2010; McGann et al., 2013). Activity is the process of making meaning between an autonomous agent (e.g., a soccer player) and the environment. By actively and asymmetrically regulating the conditions of the exchange with the environment, he/she builds meaning and enacts her *own-world* (Di Paolo et al., 2010). This own-world is how he/she experiences her own coupling with the environment in the moment (Thompson, 2007) that is, through what is, at that very moment, relevant to him/her in relation to his/her activity. For instance, what is he/she trying to do? What is drawing his/her attention? What is he/she feeling? What made his/her decide something? The situated experience lived by agents is therefore not considered as epiphenomenal, as in other theoretical approaches (e.g., Blickensderfer et al., 2010; Araújo and Davids, 2016), but instead requires phenomenological investigation (Varela et al., 1991; Thompson, 2007). The methods used in this approach are retrospective phenomenological interview techniques that can be brought together under the *first-person* approach method (e.g., Varela and Shear, 1999), in the aim of capturing team members' lived experiences at the level of their prereflective consciousness in situation through verbal description (Legrand, 2007). An enactive phenomenological analysis always gives primacy to individual subjectivity and then describes the team coordination. The analysis describes how players' experiences are arranged and then determines how these arrangements are adjusted over time (e.g., Poizat et al., 2009; Bourbousson et al., 2012; R'Kiouak et al., 2016; Gesbert and Durny, 2017).

The aim of the present study was to describe how soccer players adjusted their activity online to the need for collective behavior during competition. To do so, we used an enactive phenomenological approach to explore how the players' lived experiences were linked in the active regulation of team coordination during offensive transition situations.

## Methods

### Setting and design of the study

The present study was carried out in collaboration with the Performance Unit of Stade Rennais Football Club (a top tier French professional soccer club) throughout one season. The aim was to describe and better understand the ongoing interpersonal coordination during offensive transition situations. These situations are defined as a passage of play in which a team switches from defense to offense following a change in ball possession. In the offensive phase, the team's aim is to create and exploit open areas in order to penetrate the opponents' defense and ultimately open up opportunities to score a goal (e.g., Grehaigne et al., 1997; Bangsbo and Peitersen, 2004). In contrast, in the defensive phase, the team's aim is to deny time and space to the opponents with the ball in order to prevent their goal scoring opportunities (e.g., Grehaigne et al., 1997; Bangsbo and Peitersen, 2002).

For a number of technicians, the fast transition from defense to attack is one of the keys to success in modern soccer (e.g., FIFA, 2014). Coaches' analyses of the latest international competitions have taken note of several strategies that teams use to gain the ball and then attack the opponent goal (FIFA, 2010, 2014; UEFA, 2012). In the defensive phase, for example, the team might go after the opponent players in the most forward positions on the field and then aggressively put pressure on the ball carrier and his nearby teammates through well-coordinated horizontal and vertical movements. Once the ball is recovered, quickly moving to the opponent's midfield and split-second timing of the last pass seem to be the crucial next steps in the counterattack. To summarize, a soccer team must coordinate its actions to regain and quickly move the ball into the scoring zone.

Although coaches tend to consider the offensive transition a crucial moment in high-level competitive soccer, few studies to our knowledge have examined how players in competition experience this situation in real life. Such situations usually involve many players (a) sharing *a priori* a mutual objective (i.e., win the match), (b) having few opportunities to explicitly communicate about the future action, and (c) having little time to exploit open areas after recovering the ball in order to score. We therefore assumed that this setting would offer an opportunity to enrich the current perspectives on team coordination in a dynamic task context (Fiore and Salas, 2006).

### Participants and procedure

Fifteen French male soccer players and their coach volunteered for this study. The participants were 17 years old at the time of the study (*M* = 17.40 years old, *SD* = 0.3) and had all been playing soccer for 10 years. All the players had played and trained together for at least a year and a half (*M* = 3.75 years, *SD* = 1.94). They played in the top tier of France's under-19 category. This study was carried out in accordance with the Declaration of Helsinki. It was approved by a local Institutional Review Board of the Rennes 2 University. The players were informed of the study's purpose and were told that participation was entirely voluntary. Before the study began, players, their families, and the principal researcher approved a protocol agreement that described the study's purposes in detail and ensured player confidentiality (i.e., players were given pseudonyms). More precisely, players and their families provided written informed consent.

A Stade Rennais staff member filmed eight championship matches from the stand, mainly using a wide-angle shot focusing on the player on the ball. These matches were the material from which the offensive transition situations were extracted. This extraction process was carried out by the first author, who also holds a Union of European Football Associations (UEFA) A coaching license. Each offensive transition situation met two criteria: (a) ball recovery occurred between the halfway line and midway into the opponent's half and (b) the players had to have an opportunity to attack their opponent's goal. A total of eight offensive transition situations (defined in the following sections as S1, S2, up to S8), each lasting an average of 20 s, were extracted.

### Data collection

Two types of data were gathered: (a) continuous video recordings of the players' behaviors during competition and (b) verbalizations from post-match interviews.

A Stade Rennais staff member filmed eight championship matches from the stand, mainly using a wide-angle shot focusing on the player on the ball. This gave a continuous view of all the players involved in the offensive transition situations. By involved, we mean a player who participated in winning the ball back and the subsequent attack on the opponent's goal, either as the player on the ball or a player offering him a pass option to move the ball toward the goal. In the present study, the eight offensive transition situations involved two or three players (i.e., six situations with three players and two situations with two players). Once an offensive transition process was identified, elicitation interviews were conducted with the players involved.

Verbalization data were gathered from the elicitation interviews carried out with the players involved in each offensive transition situation. These interviews were conducted 48 h after competition and were preceded by a brief self-confrontation interview (e.g., Hauw and Durand, 2007) that consisted of showing the player the video of the extracted situation that had allowed us to identify the units of activity he had experienced from his own point of view (Zacks and Swallow, 2007; Kurby and Zacks, 2008):

“…When I saw Phil get the ball, I knew he was going to pass it to Jim and then as soon as I saw how Jim was oriented, I knew that he was going to pass it to me…He usually plays to one player, often with a deviation so I got ready by moving up…There I hesitated to make a direct kick…I wanted to move up closer and after I moved off to the side…” (Flynn).

These units of activity were then subjected to in-depth investigation during the elicitation interview, which is a technique for questioning a subject (Vermersch, 1999, 2012). The technique is designed to guide a person in recalling a given experience by redirecting his attention to specific aspects of an experience so that he can then precisely describe it (Petitmengin, 2006; Vermersch, 2009; Valenzuela-Moguillansky, 2013). The elicitation interview has been used in cognitive (e.g., Lutz et al., 2002), clinical (e.g., Petitmengin et al., 2007), and sports (e.g., Villemain and Hauw, 2014; Gesbert and Durny, 2017) research. It is used to access detailed phenomenological reports of an individual's past experience (e.g., Varela and Shear, 1999; Depraz et al., 2003; Petitmengin et al., 2013; Olivares et al., 2015).

The process of carrying out an elicitation interview can be described as four main steps (e.g., Petitmengin, 2006; Vermersch, 2012): (a) the selection of a past experience, (b) the evocation of this experience, (c) the description of the diachronic dimension of the experience (i.e., the flow of experience that is the chaining of activity units), and (d) the deepening of the experiential aspects that characterize each unit of the activity (for an illustration, see Valenzuela-Moguillansky, 2013). In the present study, the first researcher selected the past experience. Indeed, it was important to have all the players involved in a given extracted offensive transition situation provide descriptions of their lived experiences.

Therefore, the first researcher prompted each player to describe his lived experience during the extracted offensive transition situation. To do so, he led the player toward an evocation of his own past experience as if he were reliving it. This was achieved by helping him to rediscover the spatio-temporal context of the experience (when, where, with whom?) until the past situation was more present than the interview situation and the player was relating to this past experience. For example, the interviewer sometimes used questions about the spatio-temporal context of the experience to which the player could not reply without referring to the past situation (e.g., When you're repositioning yourself in the team's defensive line, what are you concentrating on?; see Petitmengin, 2006, for further details). Last, the interviewer was sensitive during the elicitation interview to behavioral indicators (e.g., the use of the present tense, a slowing of the word flow, the shifting and unfocusing of the eyes…) that indicated how the player was relating to his past experience. Once he was in state of evocation, the interviewer used the physical and/or mental actions that the player had carried out throughout the specified situation as a guide for questioning (e.g., Petitmengin, 2006; Vermersch, 2009, 2012; Valenzuela-Moguillansky, 2013). After asking him about the temporal evolution of his actions (i.e., And then…what are you doing? What are you thinking about?) and the different stages of his experience, the interviewer guided him to direct attention to finer levels of the experience in each stage on the basis of five other experiential categories: objectives (i.e., What are you trying to do?), attention (i.e., What are you concentrating on?), expectations (i.e., What are you expecting?), projections (What are you expecting will happen?), and mobilized prior knowledge (i.e., What kind of situations do you feel you are in at this moment? Do you recognize the feeling in this situation? Is it new?).

The first researcher, who had been trained in elicitation interview techniques and had gained considerable experience, conducted a total of 22 interviews. These lasted 30–45 min, were video-recorded, and then were transcribed in their entirety.

### Data processing

The video recordings were reviewed to create an inventory of the players' movements during the unfolding situations. The verbalization data were processed in four steps: (a) reconstructing the diachronic and synchronic dynamics of the players' lived experiences (e.g., Gesbert and Durny, 2017), (b) synchronizing and connecting each players' units of activity (e.g., Bourbousson et al., 2015), (c) describing the regulation modes enacted by the players, and (d) comparing the occurrences of the collective regulation modes in relation to the moment in the situations.

### Reconstructing diachronic and synchronic dynamics of the players' lived experiences

The first stage consisted of describing each of the players' lived experiences of the offensive transition situation. To do so, we used the *semiose* part of the psycho-phenomenological framework (Vermersch, 2012; Petitmengin, 2014) that corresponds to the players' sense-making process in situation (e.g., Varela et al., 1991; Di Paolo et al., 2010; McGann et al., 2013). First, from each player's descriptive statements we reconstructed the stream of his lived experience by identifying the succession of linkages between action and situation (i.e., unit of activity) considered at the level of what he enacted at the phenomenological level. These characterized the player's step-by-step experiences during offensive transition situation:

“…Jim passes to me. I control the ball and then speed up toward the goal. I see that the goalie is advanced and I think about making a lob shot. Then I realize that I'm a little far and that I can move up closer. I speed up and then I feel an opponent behind me. I think to myself that at that speed, it's going to be kind of complicated to finish…” (Flynn).

Second, we characterized the synchronic dimension of each unit of activity. To do so, we used six experiential categories: the player's objectives during the phase of play (O), the motor or mental actions carried out by the player to achieve his objective (A), the sensorial attentional content that was significant at the player's level of perception (S.Att.c), the player's expectations about the possible actions that his opponents or teammates might make (E), the player's projections about integrating his action with a teammate's action (P), and the knowledge used or built during the player's action (K). The player's statements were thus gradually assigned to these different categories. The interactions between these different categories enabled us to coherently reconstruct the player's lived experience in its synchronic dimension. To facilitate the assignment of categories, we used the video recordings to create an inventory of each player's movements and provide us with an extrinsic description of the action taking place; during the interviews, we also insisted on the coherent organization of the collected category information (see Table 1).

**Table 1:** **Illustration of a player's unit of activity at a given moment of the situation**.

**Extrinsic description**	**Phenomenological contents**
The left-back defender has the ball. He passes it to right-back defender.	(S.Att.c) The opponent player to my left has the ball—I'm a little in front of the half-way line (O) Be lined up with my teammates (E) Don't let anyone through (A) Look around at my teammates (S.Att.c) Arnold is on my left—Phil is pretty close—Jim is in front of me a little off to the side

*A, action; O, objective; E, expectation; S.Att.c, sensorial attentional content*.

These data were then used to identify the regulation modes enacted by the players. We particularly took into account the *objective*^1^ category because the objective circumscribes a players' activity in a given situation and thus provides access to the meaning the player is enacting at any instant.

### Synchronizing and connecting each player's units of activity

To describe and analyze how each player adjusted his activity with regard to his teammates (i.e., team coordination), we used a procedure for synchronizing the players' experiences (e.g., Bourbousson et al., 2015; R'Kiouak et al., 2016; Gesbert and Durny, 2017) and focused on the objective category. The players' units of activity were thus connected by presenting them side-by-side in chronological order (see Table 2).

**Table 2:** **Illustration of a collective unit of activity at a given instant of an unfolding situation**.

**Extrinsic description**	**Phenomenological contents**		
	**Flynn**	**Jim**	**Phil**
The left-back defender has the ball. He passes it to right-back defender.	(S.Att.c) The opponent player to my left has the ball—I'm a little in front of the half-way line **(O) Be lined up with my partners** (E) Don't let anyone through (A) Look around at my teammates (S.Att.c) Arnold is on my left—Phil is pretty close—Jim is in front of me a little off to the side	(S.Att.c) The left-back defender has the ball (A) I think that I shouldn't stay in front alone **(O) Return to the defensive block** (A) Move back to midfield (S.Att.c) In front of Phil and Flynn who form part of a line of 4 midfielders,	(S.Att.c) The left-back defender has the ball (A) Look to where my immediate opponent is (S.Att.c) He's pretty far from the action **(O) Back off from my direct opponent so the opponent ball carrier can make the pass** (S.Att.c) The ball carrier decides to get the ball out (E) He's going to move the game to the other end

*A, action; O, objective; E, expectations; S.Att.c, sensorial attentional content. Noted in bold are team members' objectives at the given moment when the given participant is acting*.

This connection was made using an extrinsic description of the unfolding situation provided by the video recordings. Once the players' units of activity were step-by-step connected, each connection was viewed as a collective unit corresponding to which and how individual objectives were linked at a given moment (e.g., Bourbousson et al., 2015; Araújo and Bourbousson, 2016). Each time one player experienced a change in his activity—in this case, a change in the pursued objective—a new connection arose and a new collective unit was identified. Seventy-five collective units were identified throughout the eight offensive transition situations.

### Description of the regulation modes enacted by the players

The third step was to characterize how each player experienced the adjustments made with respect to his teammates and opponents. The objective category circumscribes the players' activity by taking into account any instant meanings they are enacting. Each collective unit of activity was clustered using the related objectives within the three categories of regulation modes—local (L), global (G), and mixed (M). Local mode took into account how a player adjusted his activity based on information from the immediate environment (e.g., behaviors of nearby teammates/opponents) or on a more distant one-on-one play between a teammate and an opponent. Global mode took into account the adjustment of activity based on information about the collective organization of a part of the team (e.g., the line of midfielders). Mixed mode described the adjustment of activity based on the actions of a nearby teammate/opponent and a more distant teammate/opponent (Gesbert and Hauw, 2017). These collective units were matched to collective regulation modes that enabled us to account for the relationships between individual player's experiences across the unfolding situation (see Table 3).

**Table 3:** **Illustration of a collective regulation mode at a given instant of an unfolding situation**.

**Extrinsic description**	**Collective regulation mode**		
	**Flynn**	**Jim**	**Phil**
The left-back defender has the ball. He passes it to right-back defender.	G (GLOBAL)	G (GLOBAL)	M (MIXED)

### Comparing the occurrences of these collective regulation modes in relation to the unfolding time of the offensive transition situations

The 75 collective regulation modes were then compared in order to identify typical patterns. This comparison was carried out based on the typical phases that were present in all the offensive transition situations with reference to the coaches' analysis (FIFA, 2010, 2014; UEFA, 2012). These indicators were as follows: putting pressure on the opponent ball carrier, recovering the ball, and passing through the opponent's midline and the end of the situation. Table 4 describes the collective regulation modes for each situation and for each portion of the offensive transition situation (e.g., from the throw-in to putting pressure on the opponent ball carrier).

**Table 4:** **Number of collective regulation modes for each study situation and each period characterizing them**.

**Typical phases**	**Situations**								**Number of collective regulation modes/period**
	**S1**	**S2**	**S3**	**S4**	**S5**	**S6**	**S7**	**S8**	
Throw-in—Put pressure on the opponent ball carrier	3	2	2	3	2	1	1	1	*15*
Put pressure on the opponent ball carrier—Recover the ball	2	2	3	2	1	3	3	2	*18*
Recover the ball—Push through the opponent's midline	2	2	1	1	1	2	2	3	*14*
Push through the opponent's midline—End the situation	7	3	7	4	1	4	1	1	*28*
Number of collective regulation modes/situation	14	9	13	10	5	10	7	7	75

### Data reliability

Several measures were taken to ensure the validity of the data. The first two authors, each experienced at conducting qualitative research independently, coded 20% of the data transcripts independently to identify the unit of activity and the first author then coded the rest. Similarly, the first and third authors coded 50% of the units of activity in the collective regulation modes. By proceeding with this double coding, we were able to endure agreement rates of, respectively, 80 and 95%. A third coding session was conducted for each of these two coding parts to reach consensus for the disagreement.

## Results

A detailed analysis of the collective regulation modes enacted by the players during the offensive transition situations identified four typical patterns of collective regulation modes between teammates. These patterns were labeled as follows: (a) reorganization in play formation, (b) adaptation to actions of putting pressure on the ball carrier, (c) availability to get the ball out of the recovery zone, and (d) shoot for the goal.

### Reorganization in play formation

The main collective regulation modes that the players enacted in the first part of the offensive transition situations were G.G.G. (46.6%) and G.G.M. (33.3%; see Table 5). At the beginning of these situations, the players were not in positions typical of this type of situation due to game circumstances. They thus attempted to reposition themselves in relation to their teammates, as illustrated by the following verbatim:

“…The opponent goalie has the ball…I'm back in the block with the others…” (Jim).

“…The goalie has the ball in his hands. I reform the block…I put myself with the right midfielders” (Arnold).

**Table 5:** **Collective regulation modes enacted by the players for the pattern ***Reorganization in play formation*****.

**Collective regulation modes**	**G.G.G**.	**G.G.M**.	**G.M.M**.
Number	7	5	3
Frequency	46.6%	33.3%	20.1%

*G, mode of global regulation; M, mode of mixed regulation*.

This activity of repositioning was based on an awareness of the positions of several of their teammates, which they described as a line of players (e.g., be lined up with the other teammates in the midfield) or a defensive block (e.g., back in the block). These adjustments were thus encoded within a global regulation mode.

In contrast, some of the players were in an appropriate position at the same time. They kept an eye on the opponent ball carrier in order to assess his possibilities to act, without, however, neglecting their proximal opponent that is, the one they had defensive responsibility for. The following verbatim illustrates this enaction:

“…I'm in position with my teammates…My direct opponent in right next to me. I'm waiting to see how it's going to go for the opponent ball carrier. I don't think he can go forward in dribbling since there are so many players in front of him…He has the option of playing it long, but he's not used to that. He can maybe pass to the lateral right-back player because Arnold is a bit too much off to the side…but since he got the ball out cleanly enough, I'm leaning more toward an inside game with the defensive midfielder” (Flynn).

Their adjustments were encoded within a mixed regulation mode by combining several areas of local information (e.g., areas linked to the proximal opponent and the opponent ball carrier).

The last collective regulation mode identified was G.M.M. (20.1%). It was composed of two units of activity characterized by a mixed regulation mode.

### Adaptation to actions of putting pressure on the ball carrier

The main collective regulation modes that the players enacted between putting pressure on the opponent ball carrier and ball recovery were M.M.M. (33.3%) and M.M. (33.3%), as described in Table 6.

**Table 6:** **Collective regulation modes enacted by the players for the pattern ***Adaptation to actions of putting pressure on the ball carrier*****.

**Collective regulation modes**	**M.M.M**.	**M.M**.	**M.M.L**.	**M.L**.
Number	6	6	4	2
Frequency	33.3%	33.3%	22.3%	11.1%

*G, global regulation mode; M, mixed regulation mode; L, local regulation mode*.

Once repositioned in the team's defensive configuration, the players all had specific positions on the field (i.e., the defensive block was in place, with short distances between players both across and down the field). While checking on the opponent ball carrier's possibilities to act (e.g., the opponent ball carrier cannot play with a specific teammate), they adjusted to his behaviors as well as to the behaviors of their direct opponents, as illustrated by the following verbatim:

“The central defender takes the ball. I see the other opponent on my left, I don't think he'll be able to reach him. I especially look at the one in front of me, he's not too well-oriented. He's facing the ball carrier. I'm close enough, at a fair distance. I position myself so he passes to the opponent as far down as possible and the defender thinks he'll have time to give it to him…the midfielder is also going to think that he has the time to take the ball” (Alan).

Based on their positions on the field, the players tried to reduce and/or manipulate the opponent ball carrier's possibilities to act. For example, the player situated in the most forward position sought to prevent the opponent on the ball from passing to his teammate to his right. To do this, his activity was organized around two areas of local information.

Our results also indicated that the last collective regulation mode identified in six of the eight situations was characterized by a local regulation mode (M.M.L. or M.L.). This regulation mode was enacted by the player nearest to the opponent ball carrier, as illustrated by the following verbatim:

“The central defender passes him the ball. It's good, I can go now. I go full charge at him. He gets the ball with no change in rhythm. I feel him as soft, not too confident. I'm careful not to be eliminated. When I'm right next to him, I try to slow up a little but not too much…” (Alan).

After progressively getting closer of the opponent ball carrier for a one-on-one, he adapts his activity to the ball carrier's behaviors to regain the ball or limit his range of possible actions.

### Availability to get the ball out of the recovery zone

The collective regulation modes enacted by the players after regaining the ball were mainly L.L.L. and L.L. (64.3%; see Table 7). After regaining ball possession, the new ball carrier attempted to quickly play forward and find a fast solution to get the ball out of the recovery zone and eliminate any nearby opponents. He thus adjusted his activity only in relation to local and proximal information. His teammates adjusted their activity in relation to him and their proximal opponent.

“I have to find a solution and there I see Jim who's available up ahead” (Arnold, ball carrier).

“I have to have a solution for Arnold and then I make a decision because my defender is starting to manage things far into the opponent's half” (Jim, ball carrier's teammate).

**Table 7:** **Collective regulation modes enacted by the players for the pattern ***Availability to get the ball out of the recovery zone*****.

**Collective regulation modes**	**L.L.L**	**L.L**	**L.L.M**
Number	4	5	3
Frequency	28.6 %	35.7%	35.7%

*M, mixed regulation mode; L, local regulation mode*.

The third collective regulation mode enacted by the players was L.L.M. (35.7%). Due to his position on the field, the third player was out of the immediate visual field of his teammate on the ball. He was interested in information about the ball carrier and/or another teammate, as well as proximal opponents, thus describing an interest in several areas of local information (i.e., a mixed regulation mode).

“I see Zack intercept the ball. At that moment, I back off because I see that Jim has come to the inside. He's coming to help out. As soon as Jim sees a teammate alone, particularly me I think, he likes to play with just that player to speed up the game. I back off so I can be there while still keeping an eye on the sideline with the opponent's defenders” (Flynn).

### Shoot for the goal

The collective regulation modes enacted by the players after moving the ball away from the recovery zone were mainly L.L.M. (53.6%), L.L.L. (14.3%), and L.L. (14.3%; see Table 8). The new ball carrier wanted to attack the opponents' goal as quickly as possible. His proximal teammate situated in his field of vision was therefore the best solution to move forward. The ball carrier and this teammate thus continued to organize their activity in relation to local information. They adjusted their behaviors mutually and in relation to their proximal opponent. The following verbatim extract illustrates these enactions:

“I pass the ball and make sure it ends up with Arnold…and then I signal to get it back …” (Zack).

“Zack passes me the ball…as soon as he's done this, he starts running deep into the opponent's half between the two center players…” (Arnold).

**Table 8:** **Collective regulation modes enacted by the players for the pattern ***Shoot for the goal*****.

**Collective regulation modes**	**L.L.M**.	**L.L.L**.	**L.L**.	**L.M.M**.	**L.M.G**.
Number	15	4	4	2	3
Frequency	53.6%	14.3%	14.3%	7.1%	10.7%

*G, global regulation mode; M, mixed regulation mode; L, local regulation mode*.

In view of his position on the field (e.g., out of the immediate visual field or too far from his teammate on the ball), the third player instead tried to prepare his future call for the ball or to get into position to wait for the defensive phase, as illustrated by the following verbatim:

“Zack (ball carrier) doesn't pass me the ball. I begin to call for it in front of me, where there's no one. I run toward the goal and I bring along a defender by passing in front of him, in fact …” (Stuart).

To do this, he paid attention to his ball-carrying teammate and/or his other teammates, as well as other opponents present in the area where he wants to go, realizing that both are interesting areas of local information (i.e., mixed mode of regulation).

The third player was also able to continue trying to interact with the teammate on the ball. He thus only adjusted his behaviors using the L.L.L. collective regulation mode.

The players enacted two other collective regulation modes during the attack (L.M.M. and L.M.G.). These modes were characterized by the attacking ball carrier, who wanted to finalize the attack. He only adjusted to the proximal opponent's behaviors, with a local mode of regulation, as illustrated by the following verbatim:

“.…Jim passes to me. I control the ball and then speed up toward the goal. I see that the goalkeeper is advanced and I think about a lob shot. Then I realize that I'm a little too far away and that I can move up closer. I speed up and then I feel an opponent behind me. I think to myself that at this speed, it's going to be kind of complicated to finish…I choose to get out…” (Flynn).

The ball carrier's teammates tried to either accompany him or position themselves in the block in anticipation of a future play.

“There, I can see that Tom made a difference. I get closer to the goal but not at a real good pace, I'm kind of in the axis of the field. I'm supporting Tom's action. A defender gets closer to Tom, it's the one I had at the beginning…” (Stefen)

“I'm now a little far away to call for the ball…I'd rather get into the defensive block in case we lose the ball” (Angel)

## Discussion

In this study, we describe how individual soccer players adjusted their activity to the need for team behavior (i.e., What was the constant focus in the adaptations actively made by the players?) by analyzing phenomenological data. These data were related to the players' lived experiences throughout multiple offensive transition situations. We were thus able to characterize the pattern of collective regulation modes enacted by the team in order to coordinate. These results are discussed in two parts. First, our results suggest that team coordination may be grasped as a fluctuating phenomenon that can be described through typical patterns of collective regulation modes. Second, our results point out that soccer team coordination is supported by several processes of regulation at the level of the players' local couplings. Among these regulation processes, we propose a new mode of interpersonal regulation.

### Team coordination as a succession of patterns of collective regulation modes

Our results identified four patterns of collective regulation during the offensive transition situations. These patterns reflect the way the players adjusted their activity at the level of activity that was meaningful for them.

The first pattern describes the reorganization of the team at the beginning of the offensive transition. Due to the previous situation of ball possession, the team was disorganized at this instant. Some players were not in the positions they would normally be in to recover the ball. To reposition correctly, they intuitively adjusted their activity by aligning in a defensive block or a line of players that was re-forming (i.e., global regulation mode). These results suggest that the players' activity was directed toward a global mode of regulation that they were all bringing about (Bourbousson and Fortes-Bourbousson, 2016). To get into position from this initial phase of disorder and enact the global regulation mode, they were sensitive to information about the line of players or the defensive block. This mode can also be described as attractive because the players actively sought its emergence, which in return directly supported their adaptive activity.

This phase of team reorganization was prior to the more crucial activity of ball recovery. Once the team's defensive configuration was set up, our results indicated the emergence of a second regulation mode. According to their position on the field, the players sought to gather information about both the opponent ball carrier's possibilities for action and their immediate opponent in order to restrain (e.g., block a pass) or conversely encourage (e.g., let a pass occur) their opponent's activity. They were more tuned in to the opponent ball carrier's actions and those of their direct opponent because their respective activities were above all organized around putting pressure on the ball carrier. The modes of adjustment enacted by the players were therefore mixed. These results indicate that the players modulated their activity by spontaneous adjustments to contextual information that they were sensitive to rather than by referring to pre-established actions (Blickensderfer et al., 2010; Eccles and Tran Turner, 2014).

After ball recovery, a third team regulation pattern emerged. The new ball carrier's teammates promptly became available to help get the ball out of the recovery zone. They were thus more tuned in to information about the ball carrier's behavior, as well as that of opponents in this area of the field. Since the information they were sensitive to came from their proximal environment, the players enacted local regulation modes. Typically, these regulation modes match those identified by the ecological dynamics approach (Araújo and Davids, 2016; Passos et al., 2016), despite being related to individual lived experience. The players engaged in exploratory activity to look for and find satisfactory solutions in order to be available for their teammate's attack, while also facing dynamical environmental constraints (e.g., variations in interpersonal distance from teammates and/or opponents).

Once the ball was taken out of this area, the ball carrier and one of his teammates continued to mutually adjust by adopting local regulation modes for a quick attack on the opponent's goal. The modes were local because the transient information came from their proximal environment. The third player either adjusted to the current ball carrier's behavior and those of nearby opponents as he sought solutions via a local regulation mode or he chose to adjust to a future ball carrier's behavior and that of opponents in the area where he wanted to call for the ball via a mixed regulation mode. The behaviors of the ball carrier and his partner thus seemed to be particularly pertinent information for the players to reach their objective. Similar to the previous pattern, this mode of regulation recalls those identified in studies using the ecological dynamics approach (e.g., Passos et al., 2016).

These results highlight how soccer team members use several modes of regulation to achieve team coordination during dynamic tasks. Team coordination may therefore be described and explained as the succession of collective regulation modes. Our results describe how these modes are related to specific properties of the unfolding situation and the meanings collectively attributed to them, thereby providing a response to R'Kiouak et al. (2016), who raised questions about the parameters controlling how team members switch dynamically from one regulation process to another during an unfolding team action. Although earlier studies described a basketball team's coordination dynamics during an official match through a network analysis of the type: *who takes whom into account* (Bourbousson et al., 2010, 2015), the present study examined the adjustments enacted by soccer players in situation by adopting a phenomenological enactive analysis—that is, by gaining access to those elements that perturbed them and organized their sense-making activities.

Our results show that the players were aware of the specific regulation modes that were embedded in the game. In the situation of not possessing the ball, they showed that regulation modes chained in a relatively predictable manner: the first regulation mode referred to the players' repositioning into the team's defensive configuration (i.e., adopting a global regulation mode), which was in fact preparation for the more specific activity of recovering the ball (adopting a mixed regulation mode). The fluidity with which these regulation modes chained may reflect the habits built up through long years of training (see the next point of the discussion). Conversely, when they possessed the ball, these regulation modes chained in a less predictable way. A training challenge would thus be to develop players' capacities to switch from a global or mixed mode of regulation during the phase of non-possession to a local regulation mode after recovering the ball. This appears to be critical because the players do not know how the modes will chain. The difficulty is thus to make this transition as quickly as possible in order to maintain or even increase the imbalance provoked within the opposing team.

### Insights into regulation processes on a sports team

Our results provide insights into the nature of regulation processes enacted by soccer team members during dynamic tasks.

First, they show how the team members played in a relatively intuitive way during the phases of no ball possession: each player pursued a specific objective depending on his location on the field (e.g., prevent the pass between the player on the ball and one of his teammates). To do so, they adjusted their activity to the actions of the opponent ball carrier and his proximal opponent. In support of the results of other studies in the sports sciences (Bourbousson et al., 2010; Millar et al., 2013; R'Kiouak et al., 2016), they raise questions about the assumption of the need for *mutual awareness* to achieve team coordination (e.g., Reimer et al., 2006; LeCouteur and Feo, 2011). Our findings indeed show that the players were so absorbed in what they were doing that they paid no attention to their teammates; interpersonal regulation processes were therefore not the focus of the adaptations actively made by the players in these instants: the focus was instead on the opponent ball carrier's actions. This result suggests the emergence of a new mode of regulation between team members to achieve team coordination: the *indirect* interpersonal regulation mode. We employ the word indirect because this mode was mediated by one or more opponents and not by teammates. Further, this notion is linked to the findings of a study of a joint musical performance: Schiavio and Høffding, 2015) showed that an awareness of co-players' subjective states was not required for a string quartet's performance; the first violinist, for example, was able to play without awareness of his co-players' mental states. The authors determined that it was the quality of the music perceived by the musicians that in great part explained the co-performance. In the present study, our results indicate that the soccer players were not aware of their teammates as they coordinated to collectively recover the ball: they adjusted to each other through the behavior of the opponent ball carrier. Future research should examine what occurs in other team sports in order to determine whether this interpersonal regulation mode is specific to soccer.

Second, our results indicated a switch in regulation modes enacted by the players after regaining the ball. The ball carrier and one of his teammates mutually adjusted their activity based on a co-regulation process, whereas a third player adapted his activity in a one-directional way toward the ball carrier or the other partner. Using the terminology of Di Paolo et al. (2010), this player coordinated *to* his teammate rather than *with* him (i.e., one-sided coordination). At this moment, the players experienced a relatively high degree of temporal pressure, which can be explained by the effort being made by the opponents to reduce the imbalance resulting from the loss of the ball. Their positions and orientations on the field also made them more or less visible to all teammates, supporting the observations in basketball by Bourbousson et al. (2010). The players were thus often involved in managing direct opponents, as well as the teammates they were interacting with or expected to interact with. Factors like temporal pressure, position, or player orientation in the field may also influence the possibilities offered to team members that favor the co-regulation of their activity. Although our results do not allow us to discuss the regulation modes enacted by all the team members, they nevertheless suggest that co-regulation processes do not involve all members (Bourbousson et al., 2010; Bourbousson and Fortes-Bourbousson, 2016). They also bring new elements of knowledge to explain why an entire sports team did not need co-regulation to perform.

For instance, in the phases of no ball possession, our results describe how the players intuitively modulated their activity in relation to the behaviors of the opponent ball carrier (i.e., with an indirect interpersonal regulation mode). They indicate how the players' experiences were altered when they perceived that the defensive block was in place, suggesting typical interactions like preventing the opponent on the ball from passing to one of his teammates or giving that opponent the opportunity to pass to a teammate in a specific area of the field. Through years of training and competition situations lived by the players and the repeated patterns of player/environment interaction, they may have developed a set of dispositions to act: prereflective, practical, and tacit knowledge about how to act at this specific moment (e.g., Varela et al., 1991; Legrand, 2007). These dispositions might then have implicitly influenced their experiences and, under favorable environmental circumstances (e.g., our defensive block is in position), would then be reenacted (e.g., Hughson and Inglis, 2002; Merritt, 2015).

In addition, through extensive shared practice (i.e., 18 months of training and competition together), the players also had experienced repeated interactions with their teammates. From these repeated patterns, a collective prereflective knowledge about how to collectively regain the ball might have developed as a collective body memory (Fuchs, 2017). This memory can be defined as a set of the dispositions that characterize the members of a team, that have developed over the course of shared experiences, and that preordain the interactions between team members at a given instant (Fuchs and De Jaegher, 2009; Fuchs, 2016, 2017). These past and shared experiences (i.e., a collective body memory) are not represented throughout action; instead they are played out, actualized and reenacted in the course of the action being performed. Our results indeed describe how the players intuitively acted at these moments without the need to remember, without the need to explicitly recollect through representations what needed to be done for the team, without even taking into account other teammates. Accustomed to these game phases, the players had acquired and embedded throughout their past experiences of training and competition a set of dispositions to act that they used in a prereflective way in connection with the possibilities that were emerging from the environment. We suspect that the development of this collective body memory may be a plausible explanation of why team members do not need to co-regulate their activity in order to coordinate.

Future research should investigate the effect of training on the enaction of this type of interpersonal regulation between team members in competition. Due to the lability of high-level soccer teams (e.g., Gourcuff, 2009), we think this type of research would be relevant to shed light on the effects of training settings for the development of collective body memory.

## Author contributions

VG, AD, and DH collected and processed data. VG and DH co-wrote the manuscript.

### Conflict of interest statement

The authors declare that the research was conducted in the absence of any commercial or financial relationships that could be construed as a potential conflict of interest.

## Footnotes

^1^This notion of objective is assumed to be broad enough to deal with other concepts such goal (Schiavio and Høffding, 2015) or involvement (R'Kiouak et al., 2016).
